# Case report: Injected corticosteroids for treating leprosy isolated neuritis

**DOI:** 10.3389/fmed.2023.1202108

**Published:** 2023-06-15

**Authors:** Clarissa Neves Spitz, Izabela Jardim Rodrigues Pitta, Ligia Rocha Andrade, Anna Maria Sales, Euzenir Nunes Sarno, Nivaldo Ribeiro Villela, Roberta Olmo Pinheiro, Marcia Rodrigues Jardim

**Affiliations:** ^1^Leprosy Laboratory, Oswaldo Cruz Institute, Oswaldo Cruz Foundation, Rio de Janeiro, Brazil; ^2^Post-Graduate Program in Neurology, Federal University of the State of Rio de Janeiro, Rio de Janeiro, Brazil; ^3^Pedro Ernesto University Hospital, Rio de Janeiro State University, Rio de Janeiro, Brazil; ^4^Department of Neurology, Antonio Pedro University Hospital, Fluminense Federal University, Niteroi, Brazil; ^5^National Institute of Science and Technology on Neuroimmunomodulation, Oswaldo Cruz Institute, Oswaldo Cruz Foundation, Rio de Janeiro, Brazil

**Keywords:** leprosy, ultrasound, corticosteroid, case report, neuritis

## Abstract

One of the main manifestations of leprosy is peripheral nerve impairment. Early diagnosis and treatment are important to reduce the impact of neurological impairment, which can cause deformities and physical disabilities. Leprosy neuropathy can be acute or chronic, and neural involvement can occur before, during, or after multidrug therapy, and especially during reactional episodes when neuritis occurs. Neuritis causes loss of function in the nerves and can be irreversible if left untreated. The recommended treatment is corticosteroids, usually through an oral regimen at an immunosuppressive dose. However, patients with clinical conditions that restrict corticosteroid use or that have focal neural involvement may benefit from the use of ultrasound-guided perineural injectable corticosteroids. In this study, we report two cases that demonstrate how the treatment and follow-up of patients with neuritis secondary to leprosy, using new techniques, can be provided in a more individualized way. Nerve conduction studies in association with neuromuscular ultrasound were used to monitor the response to treatment with injected steroids, focusing on neural inflammation. This study provides new perspectives and options for this profile of patients.

## Introduction

Leprosy remains a public health problem despite efforts to eradicate it. The main manifestations are a result of the skin and peripheral nerve involvement. Neuritis may lead to nerve damage that can occur before, during, or after multidrug therapy (MDT), predominantly in multibacillary (MB) and borderline leprosy, and is commonly associated with type 1 reactions (1). It causes loss of function and can be irreversible if not properly treated. Neuritis has been defined as nerve inflammation, which presents as pain and nerve thickening in conjunction with sensory impairment, associated or not with signs of motor impairment (2–5). Leprosy was initially attributed to *Mycobacterium leprae* as the etiologic agent, but in 2008, *Mycobacterium lepromatosis* was described as a second causative species; however, there is still no consensus on its importance in the epidemiology of leprosy in humans (6). Although MDT targets the causative bacteria and promotes antigenic load reduction, corticosteroids play the most important role in the management of neuritis as an anti-inflammatory therapy (7). The optimal dose and duration of corticosteroid therapy to treat neuritis is still a matter of debate. The World Health Organization recommends a standard regimen of 12 weeks to treat acute neuritis, starting with 40 mg of prednisolone, the dose of which is reduced over the following 12 weeks. Some studies recommend longer courses for the treatment of type 1 reactions (8). Other studies have compared treatment with 40 and 60 mg of prednisone and both regimens were found to be effective; however, most of the recurrences occurred within a 6-month period after completion of the low-dose regimen (9). Van Brakel et al. (10) concluded that improvement following treatment was directly related to the severity of the nerve damage observed at the beginning of treatment. In patients who did not have neuropathy prior to acute neuritis, steroid treatment resulted in full recovery in 88% of nerves with neuropathy, but only 51% of those with chronic disease or recurrent neuropathy recovered nerve function (11).

Nevertheless, it is known that prolonged therapy and/or high doses of corticosteroids result in a high frequency of side effects, such as arterial hypertension, dysglycemia, skin rashes, and Cushing's syndrome (12, 13). Another treatment option in this patient profile is the use of high-dose intravenous methylprednisolone in the pulse therapy regimen, which can reduce the occurrence of side effects (14). Local corticosteroid injection is a common non-surgical treatment for carpal tunnel syndrome (CTS). Several studies have shown that local corticosteroid injection provides significantly greater clinical improvement of CTS than oral steroids up to 3 months after treatment, as well as an improvement in symptoms compared with a single systemic injection at 1 month follow-up (15, 16). Dammers et al. (16) used a short-acting injectable corticosteroid, 40 mg of methylprednisolone, with 10 mg of lidocaine, for the treatment of CTS. In this study, we report two cases attended at the Ambulatory Souza Araújo (ASA) Leprosy Outpatient Clinic (Oswaldo Cruz Institute—IOC, Fiocruz), a Leprosy Reference Center, in Rio de Janeiro, Brazil, in which corticosteroid injections were used for the management of focal leprosy neuritis.

## Case description

The two cases of the present study were diagnosed with leprosy according to the criteria of Ridley and Jopling (17) and were subsequently treated with MDT. They were evaluated by a dermatologist and a neurologist throughout the treatment.

### Case 1

A 24-year-old male resident of the metropolitan region of Rio de Janeiro, Brazil, was referred to the Leprosy Outpatient Clinic by the primary care service on account of diffuse skin lesions over the body, which began 10 months previously. Past medical history: yellow fever 4 years previously, was a non-drinker and non-smoker, and said he did not use medications regularly. He said there was no history of leprosy in his family. A dermatological evaluation identified >20 diffuse lesions over the body (face, upper limbs, lower limbs, and trunk) in the form of papules, nodules, and tubercles. A Mitsuda test and bacilloscopy were requested, which were positive (5 mm) and 5.25, respectively, and MDT for MB leprosy was started after classification of the borderline lepromatous form (BL/LL). The patient was assessed by a neurologist at the beginning of treatment despite not having neurological symptoms, and a neurological examination did not show nerve thickening or sensory or motor changes.

After 4 months of MDT, the patient returned to the neurologist claiming he had been suffering from paresthesia in the fourth and fifth fingers of the left hand for ~1 month. On examination, the patient had pain upon palpation in the region above the left elbow and thickening of the ulnar nerve, associated with tactile hypoesthesia, thermal and pain insensitivity, and grade 4 muscle weakness according to the Medical Research Council (MRC) Scale (18) in the hypothenar region (little finger abductor and first dorsal interosseous muscles). A nerve conduction study (NCS) showed an absence of sensory nerve action potentials (SNAPs) in the left ulnar nerve and a reduced amplitude of compound motor action potentials (CMAPs) with a conduction block and reduced motor speed in the elbow segment (Table 1). Other nerves did not show alterations upon clinical examination and an NCS. Acute neuritis of the left ulnar nerve type 1 reaction was diagnosed.

**Table 1:** Left ulnar nerve conduction values at the first and control NCS assessment.

**Site**	**Latency (ms)**	**Amplitude**	**Segment**	**Distance**	**NCV (m/s)**
**ULNAR L (M)**					
**First NCS**					
Wrist	2.76	1.49 mV	Wrist		
Below elbow	7.71	1.13 mV	Wrist- Below elbow	230 mm	46.5
Above elbow	9.69	770.00 μV	Below—Above elbow	110 mm	55.6
Arm	14.94	650.00 μV	Above elbow—Arm	110 mm	21.0
**Control NCS**					
Wrist	3.75	3.75 mV	Wrist		
Below elbow	9	3.46 mV	Wrist- Below elbow	240 mm	45.7
Above elbow	11.49	3.02 V	Below—Above elbow	90 mm	36.1
Arm	13.53	3.26 mV	Above elbow—Arm	90 mm	44.1
**ULNAR L (S)**					
5 finger wrist	0	0 mV	5 finger wrist	120	0

L, left; S, sensitive; M, motor; NCV, nerve conduction velocity; ms, millisecond; mV, millivolt; μV, microvolt; mm, millimeter.

The neuromuscular ultrasound (NMUS) evaluation showed marked ulnar thickening, measuring 24 mm^2^ at the epicondylar level and 92 mm^2^ at the supraepicondylar level [reference value (RV): 8 mm^2^], as well as homogeneous fascicular hypoechogenicity and vascular flow on the Power Doppler (Note: Power Doppler is more sensitive than the Color Doppler for detecting blood flow, but it does not provide information on the direction of flow). Ultrasound-guided local injection of 40 mg methylprednisolone with lidocaine was performed every 2 weeks above the elbow groove where the ulnar nerve was most damaged.

**Figure 1:**
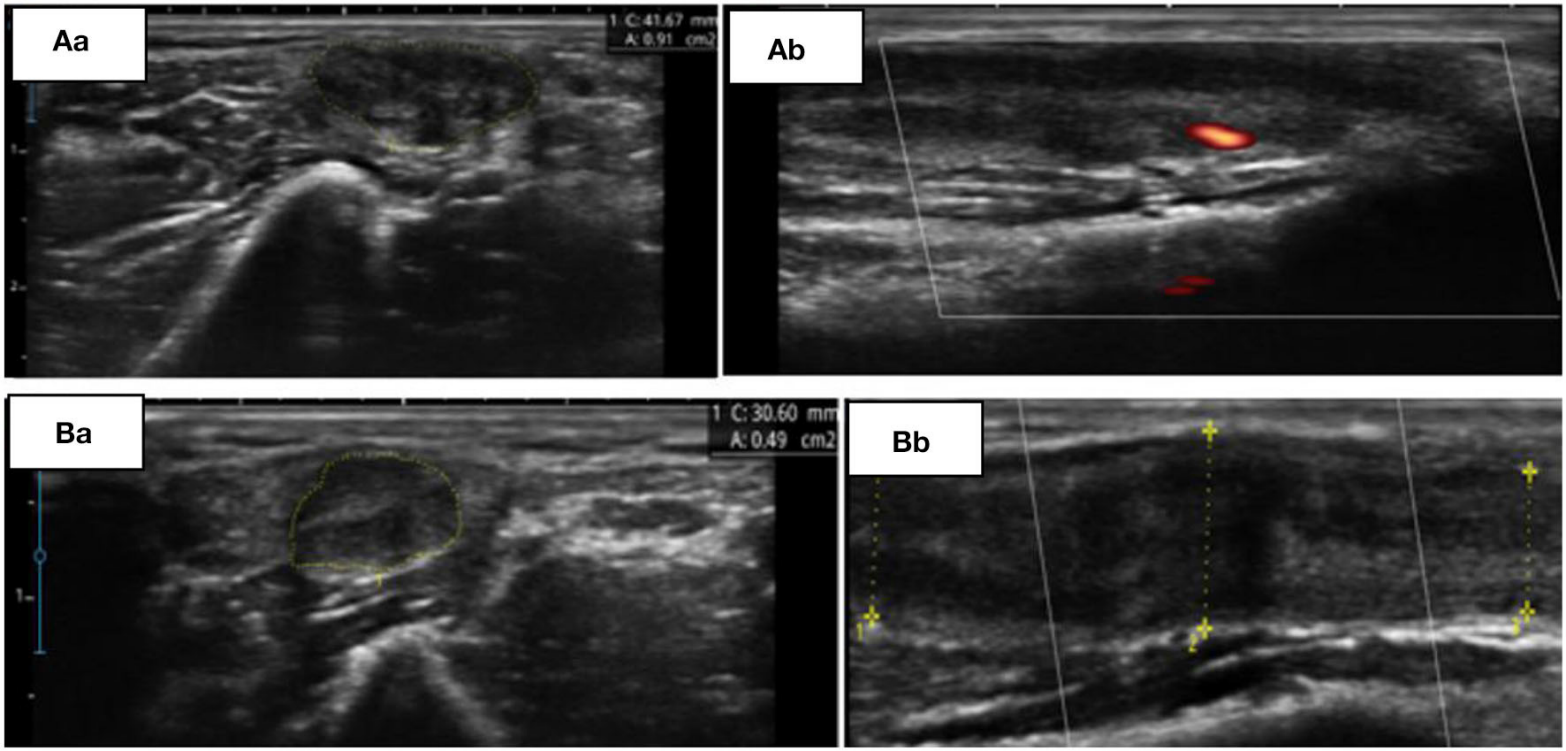
**(A)** Initial findings from ultrasonography (USG). Transverse **(Aa)** and longitudinal **(Ab)** view of the left ulnar nerve at the supraepicondylar region. **(B)** Control USG, 60 days later. Transverse **(Ba)** and longitudinal **(Bb)** view of the same nerve at the supraepicondylar region.

The patient was followed up in neurological appointments every 2 weeks, and after 2 months, the patient was reassessed and there was no complaint of paresthesia or pain upon neurological examination, an MRC grade 5 was determined in all muscles, and a subjective reduction in neural thickening was noted. An NCS was repeated within 3 months after the start of corticosteroid therapy and an improvement in electrophysiological values was observed (Table 1). NMUS showed an improvement in echogenicity and fascicular disarray, a 50% reduction in the cross-sectional diameter at the two levels described above, and no flow in the Power Doppler (Figure 1).

### Case 2

A 52-year-old female resident of the metropolitan region of Rio de Janeiro, Brazil, was referred to the Leprosy Outpatient Clinic by the primary care service because of skin lesions and neuropathic pain in her hands for the last year. The patient was under endocrinological follow-up because of insulin-dependent diabetes, for which she was using neutral protamine Hagedorn (NPH) and regular insulin (according to blood glucose measurements) with good control. The patient was dyslipidemic, a smoker (35 packs/year), a non-drinker, and had previous contact with a brother that had leprosy. She said there was no history of leprosy in her family. Physical and dermatological examination showed 11–20 well-defined, erythematous, and hypochromic lesions, with a hypoesthetic lesion in the left upper limb. Bacilloscopy was negative and skin biopsy revealed epithelioid granuloma in the dermis compatible with a reversal reaction (type 1). MDT for paucibacillary (PB) leprosy was started after it was classified as borderline tuberculoid (BT). At this time, the neurological assessment showed bilateral thickening of the ulnar nerves at the level of the elbow, thermal and painful hypoesthesia in the right ulnar nerve, and tactile hypoesthesia and thermal and pain insensitivity in the left ulnar nerve. Furthermore, muscle weakness (MRC grade 4) in the left hypothenar muscles (little finger abductor and first dorsal interosseous muscles) was observed. An NCS was performed, which demonstrated evidence of myelin lesions with secondary axonal involvement in both ulnar nerves. Ultrasonography (USG) showed ulnar nerves with thickening in the epicondylar and supraepincodylar regions and with homogeneous fascicular hypoechogenicity, as well as Power Doppler flow of the right and left ulnar nerves. There was no involvement of other neural territories observed in the initial clinical evaluation, NCS, or ultrasound. Bilateral ulnar neuritis was diagnosed and treatment with oral corticosteroids (1 mg/kg/day) was initiated, with a dose reduction of 0.1 mg/kg/day every 2 weeks until a dose of 0.5 mg/kg/day was reached, after which monthly weaning was performed until withdrawal. The total treatment time was 6 months. During this period, no significant side effects were observed, especially regarding dysglycemia, as the insulin adjustment and control were being monitored by a multidisciplinary team.

The patient was reevaluated with no signs of spontaneous pain or paresthesia, and objective sensory and motor findings were maintained. Electrophysiological examination and USG showed improvements in the ulnar parameters after 6 months.

**Figure 2:**
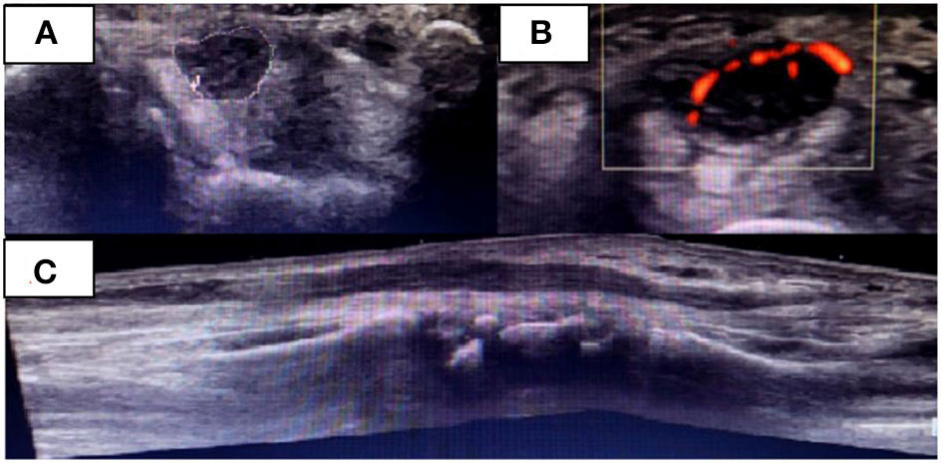
Right median nerve on the initial USG examination when median neuritis was diagnosed. Transverse **(A, B)** and **(C)** longitudinal nerve view in the carpal region. Presence of flow in the Power Doppler **(B)**.

During the follow-up of ulnar neuritis, a neurological examination suggested the involvement of the right median nerve, which was confirmed by an NCS, wherein a conduction block in the right median nerve of the forearm was observed (Table 2). USG revealed thickening of the median nerve from the level of the middle third of the pronator quadratus muscle up to the main branch of the palmar terminal branches, with a cross-sectional area 2 cm from the carpal tunnel of 32 mm^2^ and 18 mm^2^ at the carpal tunnel (RV: 9 mm^2^), and Power Doppler flow detectable in the carpal region (Figure 2). An increased signal in those regions was detected by MR neurography of the median nerve with uptake by the intravenous contrast; there were no signs of nerve compression.

**Table 2:** Right median nerve conduction values at the first and control (gray column) NCS assessments.

**Site**	**Latency (ms)**	**Amplitude (mV)**	**Distance (mm)**	**NCV (m/s)**	**Latency (ms)**	**Amplitude (mV)**	**NCV (m/s)**
**Median R (M)**							
Wrist	3.3	6.0			3.27	6.81	
Forearm	4.4	3.2	60	54.1	4.68	4.39	35.5
Elbow	9.9	3.0	180	32.6	10.53	3.72	17.1
Arm	11.1	3.0	90	78.9	12.33	4.07	61.1
**Median R (S)**							
**Site**	**Latency (ms)**		**Amplitude**		**Segment**	**Distance**	**NCV (m/s)**
3 finger wrist	0		0 mV		3 finger wrist	140	0

R, right; S, sensitive; M, motor; NCV, nerve conduction velocity; ms, millisecond; mV, millivolt; μV, microvolt; mm, millimeter.

The diagnosis of right median neuritis was made when the patient was finishing the oral prednisone treatment for ulnar neuritis. Therefore, the patient was started on 40 mg methylprednisolone and lidocaine injected around the perineurium of the median nerve over 3 months. She underwent an NCS after this period, which showed improvement in the nerve conduction values (Table 2). Additionally, there was an improvement in the USG results in terms of the nerve thickening and absence of flow on the Power Doppler. The patient remains in control of the neuropathic pain through quarterly assessments by the neurology team.

## Discussion

The clinical form of leprosy depends on the host's immune response to *M. leprae* antigenic determinants (19, 20). The evolution of neurological manifestations in leprosy is related to the clinical forms and the leprosy reactions.

Acute neuritis in MB patients has been described; however, in general, there is little inflammatory response in MB and the symptoms evolve slowly, generating a progressive symmetrical polyneuropathy (19–21), like the neuritis that occurs by complement activation (22). Additionally, silent neuritis has been described and can occur in reactions without clinical manifestations. By contrast, in PB patients, the neural involvement is more limited, occurring as a mononeuropathy or mononeuritis multiplex; patients with borderline forms are the ones who most frequently have neurological damage and complications, as they have an unstable immune response (19–21). The patient of case 2 was PB, diagnosed as having borderline leprosy, and presented median neuritis with the additional involvement of the ulnar nerve, evidencing this immunological instability that generates edema and neural compression above the entrance to the carpal tunnel.

High-resolution USG has been used to evaluate peripheral nerves. Nerves are often enlarged in leprosy patients, especially those with type 1 reactions. Lugão et al. (23) noted that the greater the thickness of the nerve, the greater the flow on the Power Doppler. It is likely that increased blood flow is the first sign of neural injury (24). In the two reported cases, it was possible to see a clear loss of fascicular morphology and neural hypoechogenicity with significant thickening in the affected nerves. The presence of flow on the Power Doppler raises suspicion of the presence of nerve inflammation, and associated with the clinical and electrophysiological findings, neuritis can be diagnosed.

The median nerve of case 2 showed significant thickening proximal to the carpal tunnel (~55% greater than the thickening in CTS) with flow on the Power Doppler. These findings are similar to the USG changes of the median nerve reported in other studies of patients with leprosy. In these studies, morphological changes and fusiform neural thickening occurring 2–5 cm from the wrist were observed, which is different from what occurs in patients with CTS (25, 26).

Patients diagnosed with leprosy may have to receive high doses of corticosteroids, and perhaps even more than once in cases of recurrent neuritis (1). The use of systemic corticosteroids is limited in patients with comorbidities and other clinical conditions, such as diabetes mellitus, cataracts, hypertension, and immunosuppressed patients. Accordingly, steroids administered at the specific point of neuritis, guided by NCS and ultrasound, can be a safe and successful strategy in these patients. An anesthetic and corticosteroid solution injected around the nerve promotes the hydrodissection mechanism and release of the anti-inflammatory and perineural analgesic medication (27–29). This therapeutic modality may be a promising alternative in cases of leprosy-isolated neuritis.

The use of injectable medications has arisen and evolved with the improvement in clinical, NSC, and imaging parameters, and may be useful for leprosy-isolated neuritis in particular. It is expected that further studies will be carried out on this method to be able to offer these patients more treatment options with the aim of reducing the definitive neurological deficits.

## Data availability statement

The original contributions presented in the study are included in the article/supplementary material, further inquiries can be directed to the corresponding author.

## Ethics statement

Written informed consent was obtained from the individual(s) for the publication of any potentially identifiable images or data included in this article. Written informed consent was obtained from the participant/patient(s) for the publication of this case report.

## Author contributions

CS, MJ, and NV: conceived the study. CS, IP, LA, and AS: performed the review of medical records. CS and MJ: analyzed the data. CS: wrote the paper and performed radiology analysis. CS, MJ, IP, LA, and AS: performed clinical evaluation. CS, MJ, IP, and LA: performed neurological and electrophysiological testing. MJ: performed several edits of the draft manuscript and supervised neurological testing. NV: performed procedures. MJ and NV: contributed to the writing of the paper. ES and RP: technical and operational support. All authors contributed to the article and approved the submitted version.
